# ﻿Chronicle of a death foretold: *Lepanthes nasariana* (Orchidaceae, Pleurothallidinae), a newly described high-Andean orchid facing a worst-case climate change scenario

**DOI:** 10.3897/phytokeys.266.161410

**Published:** 2025-11-19

**Authors:** Juan Sebastián Moreno, Angie Tatiana Herrera Cobo, Rubén Darío Palacio, Nicolas A. Hazzi

**Affiliations:** 1 Fundación Ecotonos, Cali, Colombia, Cra. 72 #13A 56, Cali, Valle del Cauca, Colombia; 2 Jardín Botánico de Cali, Fundación Zoológica de Cali, Cra. 2 Oe. #21, Cali, Valle del Cauca, Colombia; 3 Wildlife Conservation Society (WCS), Carrera 24 D No. 6 Oeste – 10, Cali, Valle del Cauca, Colombia; 4 Escuela de Biología, Facultad de Ciencias, Universidad Industrial de Santander, Bucaramanga, Santander, Colombia; 5 Fundación Ecotonos, Cali, Valle del Cauca, Colombia

**Keywords:** Biodiversity loss, conservation strategies, endemism, extinction risk, habitat fragmentation, habitat suitability, high-emission scenarios, montane ecosystems

## Abstract

Newly discovered species are increasingly found to be threatened. For some, their formal description may already foretell their extinction, a phenomenon we here term the “Nasar Effect.” This phenomenon is inspired by the tragic fate of Santiago Nasar, the protagonist of Gabriel García Márquez’s Chronicle of a Death Foretold, whose impending death is known to everyone but himself. The Nasar Effect is particularly evident in climate-vulnerable ecosystems, where species may be projected for extinction based on dramatic climate-driven habitat loss. We illustrate the “Nasar Effect” through the description of a new orchid species, *Lepanthes nasariana* (Lepanthes subsect. Breves), endemic to the cloud forests and páramos of the Western and Central Andes of Colombia, between 2,800 and 3,600 m elevation. The species inhabits mossy branches in shaded, humid environments and is most similar to *L. mefueensis*, from which it differs by its oblong-lanceolate leaves, falcate petal lobes, and narrowly ovate lip blades with an inflexed appendix, among other characters. Based on its current extent of occurrence (27,502 km^2^) and area of occupancy (12,775 km^2^), *L. nasariana* is preliminarily assessed as Least Concern (LC) following the IUCN Red List guidelines. However, species distribution models projected to 2090 under the SSP5–8.5 scenario indicate a 96% loss of suitable habitat, which would qualify the species as Critically Endangered (CR) under Criterion A3(c). Without immediate and concerted global efforts to mitigate emissions, *L. nasariana* exemplifies the potential fate of many species described from climate-vulnerable ecosystems, such as the high Andean mountains, where they may already be on a predestined short path to extinction.

## ﻿Introduction

The ongoing human-caused planetary crisis is driving an unprecedented rate of biodiversity loss, threatening ecosystems and the balance of life on Earth (Cardinale et al. 2012; Díaz et al. 2019). Alarmingly, many species face extinction before they are even discovered (Liu et al. 2022), sparking a race against time to identify, describe, and catalog new species before they vanish (Costello et al. 2013; Boehm and Cronk 2021). It is estimated that three out of four undescribed plants may already be threatened (Brown et al. 2023), given concurrent habitat loss and climate change (Boonman et al. 2024) patterns. These undescribed species are often rare endemics confined to extremely limited ranges (Pimm et al. 2014; Enquist et al. 2019). Many of them are found in mountain ecosystems, where climate change effects have been identified as one of the most significant threats (Foster 2001; Kohler et al. 2010; Alarcón Hincapié and Pabón Caicedo 2013; Schmeller et al. 2022).

Climate change impacts on mountain ecosystems extend far beyond rising temperatures, triggering a cascade of ecological disruptions. These include shifts in precipitation patterns, altered hydrological cycles, and reduced cloud cover (Helmer et al. 2019; Pabón-Caicedo et al. 2020; Mata-Guel et al. 2023; Guzmán Q. et al. 2024). Collectively, these changes heighten the risk of habitat loss and diminish the availability of suitable environmental niches for mountain species (Richards 2021; Cuesta et al. 2023). Many of these species, constrained by narrow ranges and elevation-dependent habitats, may struggle to migrate to more favorable conditions (Bell et al. 2014), with outcomes heavily influenced by species-specific environmental responses (Mamantov et al. 2021). While some species may track cooler temperatures by shifting to higher elevations, particularly in tropical regions (Freeman et al. 2021), for others the lack of higher elevations makes upward migration impossible.

Ultimately, climate change patterns pose a severe threat to the survival of highly specialized mountain species, exacerbating the biodiversity crisis in these ecosystems. This is particularly concerning in the tropical Andes, one of the most biodiverse regions on the planet, exhibiting high levels of endemism and species turnover (Myers et al. 2000; Hazzi et al. 2018). This region harbors an extraordinary richness of plant species, many of which remain undiscovered (Rahbek et al. 2019). Of particular importance are epiphytes, which constitute 30–50% of the vascular flora in neotropical mountains (Nieder et al. 1999; Kelly et al. 2004; Gómez González et al. 2017; Taylor et al. 2022) and are highly vulnerable, with an estimated 60% of neotropical epiphytes already facing extinction risk (Carmona-Higuita et al. 2024). Orchids, the most diverse group of vascular epiphytes and one of the largest plant families globally, face heightened risks due to their highly specialized habitat requirements, including narrow microclimatic tolerances and specific ecological interactions (Zotz 2013; Mondragón et al. 2015; Acevedo et al. 2020). These constraints make them particularly vulnerable to habitat loss and environmental change.

Climate change can also disrupt critical phenological events, leading to mismatches between flowering periods and pollinator activity (Robbirt et al. 2014; Hutchings et al. 2018). Although such mismatches have been documented in other plant groups, their implications for orchids remain insufficiently explored (Wraith and Pickering 2018). However, emerging evidence suggests this gap is narrowing. For instance, Watteyn et al. (2025) modeled the potential spatial decoupling between wild *Vanilla* Plum. ex Mill. species and their pollinators under climate change scenarios, showing that species with narrow pollination niches are at particularly high risk of losing reproductive success due to future range mismatches. Similarly, Karremans (2024) highlights how the uncertainty surrounding the identity and effectiveness of pollinators of *Vanilla planifolia* Andrews—combined with its low natural fruit set and climate-driven vulnerability—underscores the urgency of integrating pollinator dynamics into conservation strategies for orchids and their wild relatives.

*Lepanthes* Sw. is not only one of the most diverse genera of orchids but is also considered a megadiverse plant genus globally, with 1,196 described species (Karremans et al. 2023; Moonlight et al. 2024). Colombia is the country with the highest richness of *Lepanthes*, totaling 386 species, accounting for 20% of the Pleurothallidinae subtribe in the country (Karremans et al. 2023). Despite their wide distribution, most *Lepanthes* species exhibit high levels of endemism (Luer and Thoerle 2012), with many known from a single locality (Luer and Thoerle 2012; Pupulin and Bogarín 2012; Karremans et al. 2023), such as mountaintop slopes (Pupulin and Bogarín 2012). This ecological specialization makes *Lepanthes* highly susceptible to the effects of climate change (Tremblay et al. 1998; Crain 2012; Luer and Thoerle 2012; Pupulin and Bogarín 2012; Acevedo et al. 2020).

In this study, we describe a newly discovered species of *Lepanthes* with a narrow distribution in high-elevation cloud forests and páramo ecosystems of the Western and Central Cordilleras of the Colombian Andes. We apply species distribution models (SDMs) to assess its current and potential future distribution under different climate change scenarios. Based on these analyses, we use IUCN Red List criteria to evaluate its present and projected conservation status (Zurell et al. 2023; Perdomo et al. 2024). Additionally, using this species as a case study, we explore the broader implications of what we term the “Nasar Effect”, a phenomenon in which newly described species are predicted to face imminent extinction. This effect highlights the urgent challenges of biodiversity discovery and conservation in an era of rapid environmental transformation, underscoring the need for accelerated efforts to document and protect species before they are lost.

## ﻿Materials and methods

### ﻿Taxonomic description

Descriptions and drawings were prepared from living specimens and flowers preserved in 70% ethanol. Vegetative structures were measured from dried material, and reproductive structures from spirit material. Digital images were taken with a Nikon D750 camera and a 105 mm f/2.8 macro lens. Sketches from specimens were digitized and used to create a draft template in Adobe Photoshop® CS6. A digital composite line drawing was then made (lines and stippling) using the Procreate illustration application on an iPad 6^th^-generation tablet computer (Bogarín Chaves et al. 2019). The new species was described following standard botanical terminology (Stearn 1992; Luer and Thoerle 2012; Beentje 2016). In addition, all original descriptions of related species were consulted for detailed comparisons (Luer 1996; Dodson and Luer 2011; Luer and Thoerle 2012). The typology of the inflorescences follows the classification proposed by Rojas-Alvarado and Karremans (2024) for Pleurothallidinae.

### ﻿Occurrence and environmental data

A total of nine records were obtained for the new species, along with specimens consulted from the following herbaria: AMES, CAUP, COL, CUVC, HUA, JAUM, JBB, TOLI, and VALLE. These records provided the data used to model the species’ distribution. Environmental data were retrieved from the WorldClim database (https://www.worldclim.org; Fick and Hijmans 2017), which provides key bioclimatic variables such as temperature, precipitation, and climatic variability, including measures of seasonality and extreme conditions (e.g., temperature annual range, precipitation seasonality). To reduce multicollinearity among predictor variables, which can lead to inflated variance and unreliable model estimates, we selected variables with Pearson correlation < 0.75. In addition, variables were chosen based on their biological relevance and interpretability for the species’ ecology. For example, we retained annual mean temperature (Bio1) instead of highly correlated variables such as maximum temperature of the warmest month (Bio5), because Bio1 provides a more ecologically meaningful representation of the thermal environment. The final set of predictors included annual mean temperature (Bio1), mean diurnal temperature range (Bio2), temperature seasonality (Bio4), annual precipitation (Bio12), precipitation seasonality (Bio15), and precipitation of the warmest quarter (Bio18). These variables were used as predictors for the current period (1950–2000) and future scenarios for 2070 (2061–2080) and 2090 (2081–2100). For future scenarios, we utilized downscaled and calibrated projections from General Circulation Models (GCMs) based on AR6 (Masson-Delmotte et al. 2021) of the Intergovernmental Panel on Climate Change (IPCC) under two Shared Socioeconomic Pathways (SSPs): SSP5–8.5, representing a pessimistic scenario, and SSP2–4.5, representing an optimistic “stabilization” scenario. These contrasting scenarios were chosen to capture a range of possible future climate policies. We did not include SSP1–2.6, the most optimistic pathway, because recent analyses indicate that current global greenhouse gas emission trends more closely follow SSP2–4.5 and make the deep reductions required for SSP1–2.6 increasingly unlikely to be achieved (Hausfather and Peters 2020; UNEP 2023; Ritchie and Dowlatabadi 2024). From a conservation biology perspective, it is prudent to base conservation status assessments on realistic-to-worst-case climate trajectories rather than overly optimistic ones, following the precautionary principle. Overestimating future habitat stability could underestimate extinction risk, leading to insufficient or delayed conservation action. The modeling area was defined by considering the accessible area of the species—based on the BAM diagram (Barve et al. 2011)—over relevant time periods, encompassing historical factors associated with the species’ distribution, without explicitly considering SDMs. Based on records of the new species and the geographic and biogeographic features of the Andes (Hazzi et al. 2018), we hypothesized its accessible area (i.e., the region that the species could have reached over relevant time periods, given historical dispersal barriers and biogeographic constraints; see Soberón and Peterson 2005) in the Western and Central Cordilleras of Colombia, as well as the warm Cauca River valley.

### ﻿Distribution modeling

The distribution of the new species was estimated using the Maxent algorithm (Elith et al. 2006; Phillips et al. 2006). Models were run with random seed, maintaining the maximum number of random background points at 1,000. To assess model performance, we applied a k-fold cross-validation procedure, splitting occurrences into training and testing datasets (80% and 20%, respectively), and replicating the process 15 times. Models were evaluated using the Area Under the Curve (AUC) metric, which compares model results with null expectations using a threshold-independent measure and indicates the model’s accuracy, with values closer to 1.0 representing better performance. We averaged the AUC values from the replicates and constructed 95% confidence intervals to assess model significance compared to random expectations (AUC > 0.5). Projections of present climatic suitability to future scenarios were produced using Maxent’s default settings, and clamping maps were inspected to assess any “extrapolation” of current conditions to future scenarios. Outputs were plotted as continuous pixel values representing climatic suitability, which were subsequently converted to binary presence/absence (suitable/unsuitable) values using the minimum presence threshold. This threshold was deemed appropriate because all records were obtained and georeferenced by the authors, minimizing potential errors in georeferencing or species identification. We averaged future projections for each GCM and scenario, estimating the coefficient of variation to evaluate geographic uncertainty in our mean future predictions. Finally, changes in climatic suitability between current and future scenarios were assessed by calculating the difference between future and present in both continuous and binary models. All spatial analyses were performed using ArcGIS 10.1.

### ﻿Conservation status assessment

To assess the conservation status of the new *Lepanthes* species, we applied IUCN Red List criteria A and B (IUCN 2024). In line with Annex 1 of the Guidelines, we adopted a precautionary but realistic approach to uncertainty, documenting all assumptions, input data, and estimates of error.

For Criterion A, which evaluates population reductions based on past, present, or projected declines, we used species distribution models (SDMs) to estimate habitat loss over time. Projections were calculated to 2090 under two climate scenarios (SSP2–4.5 and SSP5–8.5). Although the strict application of Criterion A requires declines to be measured over three generations or 10 years (whichever is longer, up to 100 years), generation length is currently unknown for *Lepanthes nasariana* and most related miniature orchids. We therefore used habitat reduction as a precautionary proxy for population reduction, as recommended in the IUCN Red List Guidelines and in line with Tracewski et al. (2016). These projections are thus presented as an exploratory assessment of risk rather than a definitive Red List categorization.

For Criterion B, we estimated the current extent of occurrence (EOO) and area of occupancy (AOO) using the ConR package in R (Dauby et al. 2017). EOO was calculated using a minimum convex polygon around all known localities, and AOO was refined using the suitable habitat identified in current SDMs to improve ecological realism. These metrics were then compared against IUCN thresholds for Criterion B.

We note that our extinction risk analyses based solely on SDMs do not meet the methodological standards required for a formal assessment under Criterion E, which is typically based on population viability analyses (PVAs) or coupled habitat–population models. Consequently, results from such analyses are presented here as an exploratory evaluation of climate-driven risk, but not as a formal application of Criterion E under the Red List framework.

All spatial analyses were conducted using ArcGIS Pro 2.8 (Esri, Redlands, CA, USA) and R 4.1.0 (R Core Team 2021), with the packages ‘sp,’ ‘raster,’ and ‘dismo’ (Hijmans et al. 2023).

## ﻿Results

### ﻿Taxonomic treatment

#### ﻿ Lepanthes nasariana

Taxon classificationPlantaeAsparagalesOrchidaceae

J.S.Moreno & Hazzi sp. nov.

D3F1E630-9ADE-5B91-85C2-AAA3D76FADC2

urn:lsid:ipni.org:names:77372105-1

Figs 1, 2

##### Type.

**Colombia** • Valle del Cauca: Municipio de Cali, Vereda Peñas Blancas, PNN Farallones de Cali, Minas del Socorro, Quebrada La Española, 3115 m, 29 January 2020, *R. Galindo-T, A. Fierro, G. Rodríguez, and M. Espitia 1473* (**holotype**: CUVC; isotype: CUVC).

**Figure 1.**
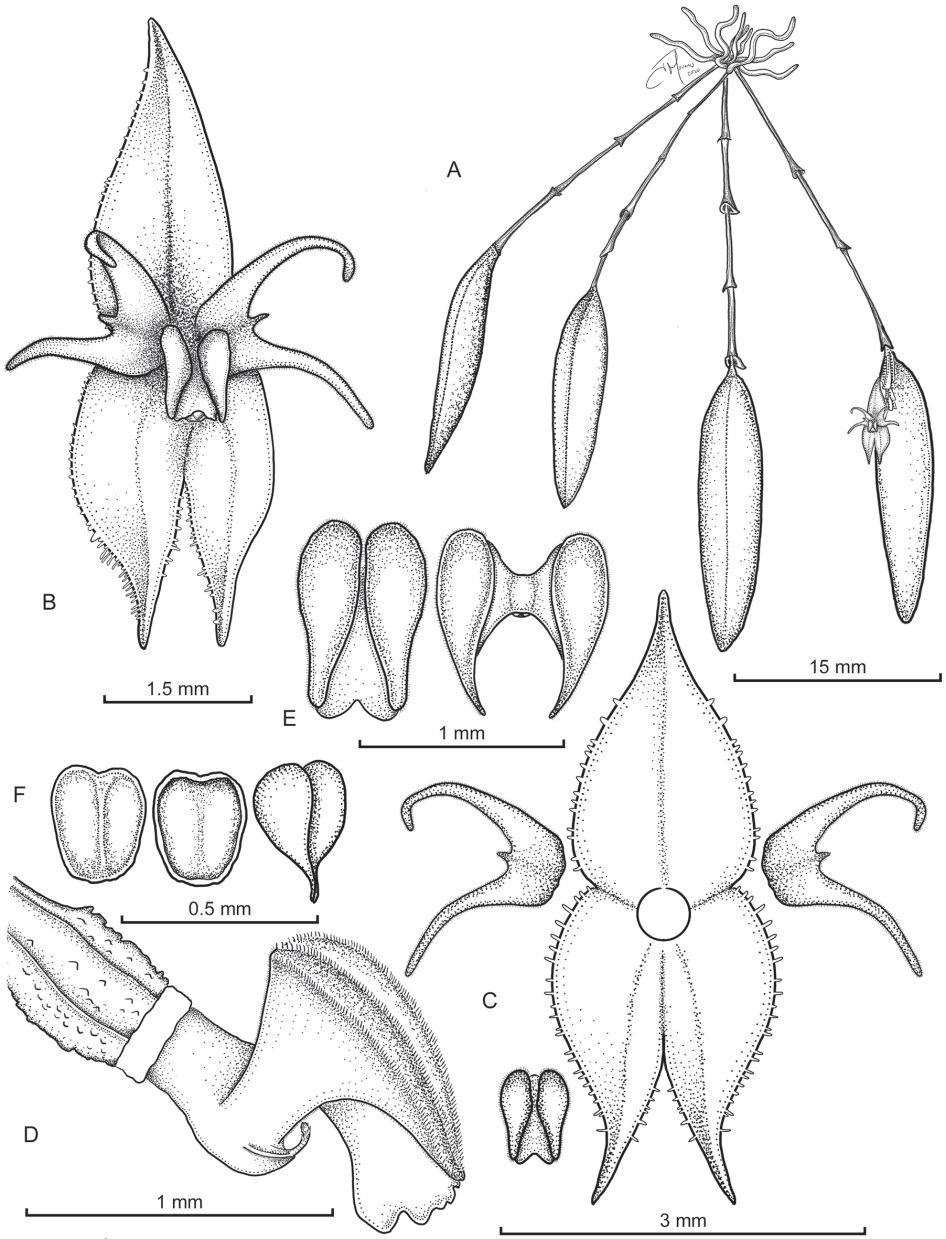
*Lepanthes nasariana* J.S.Moreno & Hazzi. **A.** Habit; **B.** Flower; **C.** Dissected perianth showing the dorsal sepal, lateral sepals, petals, and lip; **D.** Lip, column, and ovary (lateral view); **E.** Lip: left, dorsal view in natural position with the anther cap; right, expanded view showing the laminae, body, and connectives; **F.** Anther cap and pollinia: left, dorsal view of anther cap; right, ventral view showing its attachment surface and paired pollinia. Drawing by J.S. Moreno.

##### Diagnosis.

The new species is most similar to *Lepanthes mefueensis* Luer & R.Escobar, but it can be distinguished mainly by its succulent, oblong-lanceolate leaves (vs. elliptic leaves); transversely bilobed petals with both lobes narrowly triangular, falcate, and a marginal triangular midlobe (vs. lobes narrowly oblong); and the lip with blades narrowly ovate with a filiform and pubescent inflexed appendix (vs. ovate blades and a filiform, reflexed appendix).

**Figure 2.**
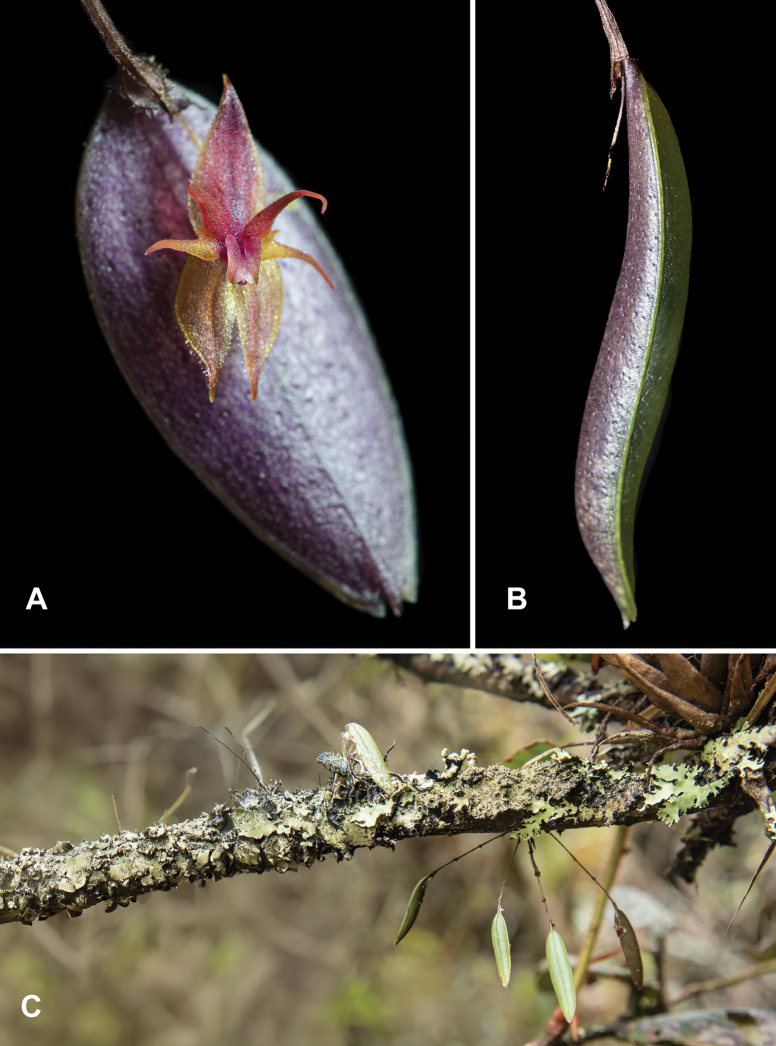
*Lepanthes nasariana* J.S.Moreno & Hazzi *in vivo*, Roncesvalles, Tolima. **A.** Flower, frontal view; **B.** Leaf, showing its thick, succulent morphology; **C.** Habit of the plant *in situ*, showing the growth form and habitat. Photographs by J.S. Moreno.

##### Description.

***Plants*** small in size, epiphytic, caespitose, up to 4.5–5.0 cm tall; roots slender, flexuous, filiform, 0.5 mm in diameter. ***Ramicauls*** slender, tight, suberect 18–23 mm long, enclosed by 4–5 acuminate, furrowed, and microscopically pubescent lepanthiform sheaths, with a dilated, ciliate ostia. ***Leaves*** purple abaxially, coriaceous, succulent, oblong-lanceolate, 15.7–20.3 × 3.3–4.7 mm, apex emarginate with an abaxial apiculum in the middle, base cuneate, contracted into a petiole 2–3 mm long. ***Inflorescence*** a congested, distichous raceme, 12–20 successively many-flowered, up to 18 mm long, including the pseudopeduncle of each multi-flowered coflorescence, held appressed to the abaxial surface of the leaf by a filiform, terete pseudopeduncle, 3 mm long, borne near the apex of the ramicaul; floral bracts purple, conical, acuminate, minutely verruculose, 0.5–0.7 mm long; pedicels terete, up to 1 mm long. ***Ovary*** terete, costate, sparsely verrucose, up to 0.7 mm long. ***Flowers*** with burgundy dorsal sepals with saffron margins, the lateral sepals saffron, slightly tinged with light burgundy along the midvein; petals with a vermilion upper lobe and saffron lower lobe with the apex tinged with light burgundy; lip blades crimson, degrading to saffron towards the apex, column crimson degrading to white near the apex. ***Dorsal sepal*** ovate, acuminate, denticulate, apex reflexed, 3-veined, carinate, 2.6–2.7 × 1.4–1.5 mm, connate to the lateral sepals for 0.5 mm. ***Lateral sepals*** ovate, acuminate, denticulate, oblique, slightly attenuate, 1-veined, 2.5–2.6 × 0.9–1.0 mm, connate for 1 mm. Petals transversely bilobed, lobes narrowly triangular, falcate, ciliate, microscopically pubescent, obtuse, 0.5–0.6 × 1.4–1.6 mm, with a filiform marginal triangular midlobe. ***Lip*** bilaminate, microscopically pubescent; blades narrowly ovate, acute, bases rounded, 0.88–0.90 × 0.24–0.31 mm, supported by cuneate connectives from near the base; body broad, adnate to the base of the column; sinus obtuse, with a filiform, oblong, pubescent, inflexed appendix, emerging from the base of the main structure, concealed in dorsal view and visible only in lateral view. ***Column*** terete, dilated, with the stigma bilobed with oblong lobes, 0.9–1.0 mm long, anther dorsal, stigma ventral. ***Anther cap*** cordate, cucullate, 0.2 mm wide. ***Pollinia*** 2, yellow, pyriform, narrowly obovoid, 0.4 mm long.

##### Etymology.

The specific epithet *nasariana* refers to Santiago Nasar, the protagonist of the novel “Chronicle of a Death Foretold” by Colombian author Gabriel García Márquez. The name was chosen in allusion to the character’s tragic fate—unaware of the threats around him, he is doomed to die prematurely. This mirrors the situation of the newly described species: although it may appear stable today, its extinction is predicted in the near future. The species is expected to undergo a “foretold death” due to the increasing frequency and intensity of extreme climatic events, driven by anthropogenic acceleration of climate change. While climate has always fluctuated naturally, it is the unprecedented speed and magnitude of current shifts—caused by human activity—that now pose a critical threat to biodiversity.

##### Habitat and ecology.

The species occurs in high Andean forests and páramos of the Western and Central Andes, extending toward the Colombian Massif. It thrives in highly humid forests with small-statured trees near streams, often dominated by abundant moss. These forests are characterized by tree species such as *Brunellia goudotii* Tul., *Hesperomeles ferruginea* (Juss. ex Pers.) Benth., *Myrcianthes rhopaloides* (Kunth) McVaugh, *Weinmannia pubescens* Kunth, and *Weinmannia rollottii* Killip. Co-occurring epiphytic orchids commonly found in these habitats include *Lepanthes intonsa* Luer, *Fernandezia myrtillus* (Rchb.f.) Garay & Dunst., *Gomphichis altissima* Renz, and *Epidendrum restrepoanum* A.D.Hawkes

##### Additional specimens examined (paratypes).

**Colombia** • **Valle Del Cauca**: Municipio de Pradera, Finca La Esperanza, sobre camino ceja que conduce al páramo de las Tinajas, 3482 m, Jul 2018, *G. Reina, I. Nicholls & H. Arenas 2651* (CUVC); • **Cauca**: Municipio de Puracé, Corregimiento de Paletará, vía Paletará – Isnos, PNN Puracé, 3100 m, July 2024, *A. Zuluaga & J.S. Moreno 6347* (CUVC); Municipio de Totoró, Corregimiento de Gabriel López, 3110 m, March 2016, *J.S. Moreno & A. Erazo 260* (CAUP); • **Caldas**: Municipio de Riosucio, Arroyo Hondo, bosque de la truchera de los Alpes vía Jardín–Andes, 2800 m, October 2021, *T. Arias, S. Vieira, E. Restrepo & D. Cadavid 711* (CUVC); • **Quindío**: Municipio de Salento, cerca de las Crestas de Salento, predio privado, 2900 m, October 2024, *E. Restrepo & S. Styles 296* (JBB); • **Tolima**: Municipio de Roncesvalles, Yerbabuena, 3366 m, November, 2018 *M. Rincón & J.S. Moreno 2350* (TOLI).

##### Additional records.

**Colombia. Antioquia**: Municipio de Urrao, Páramo de Frontino, 3413 m, August 2021, *S. Vieira & E. Dominguez* (Photo!); Municipio de Urrao, Alto del Diablo, 3584 m, November 2020, *S. Vieira & E. Dominguez* (Photo!).

##### Taxonomic notes.

*Lepanthes nasariana* belongs to subgenus Lepanthes, section Lepanthes, subsection BrevesLuer (1996), morphologically characterized by having one-veined lateral sepals and racemes in which the rachis between floral bracts exceeds the pedicels (Luer 1996). The subsection is represented by nearly 100 species in the Neotropics, with particularly high richness in the northern Andes (Luer and Thoerle 2012). In Colombia, L. subsect. Breves comprises about 40 species, most of them restricted to high Andean forests and páramos, making it a significant component of the national *Lepanthes* flora, which currently includes over 370 described species. Recent phylogenetic analyses (Arias et al. 2025) have confirmed the distinctiveness of the “Monoptera” clade, which encompasses the type species of L. subsect. Breves (*Lepanthes monoptera* Lindl.) and many other members traditionally placed in this subsection. Although morphological homoplasy is common in *Lepanthes*, the univeined lateral sepals remain a practical diagnostic feature that unites species of L. subsect. Breves for identification purposes. In Bolivia, the subsection shows an even higher relative diversity, including 24 of the 67 *Lepanthes* species (over one-third) recorded in the country, with some taxa exhibiting unusual features such as plicate or involute lateral sepals, underscoring the morphological breadth of the group across its range (Luer and Thoerle 2010).

As stated in the diagnosis, *L. mefueensis*, a species of L. subsect. Breves restricted to the Cordillera Oriental of Colombia (Norte de Santander), is the most similar to *L. nasariana*. However, *L. nasariana* can be readily distinguished by its plants with succulent, oblong-lanceolate leaves, purple abaxially (vs. coriaceous, elliptic leaves, green abaxially); ramicauls shorter, enclosed by 4–5 acuminate, furrowed lepanthiform sheaths (vs. 7–9 long-ciliate sheaths with dilated ostia); and racemes congested and up to 18 mm long, borne appressed to the abaxial surface of the leaf (vs. shorter racemes, c. 5 mm long, borne behind the leaf). Floral morphology also provides clear diagnostic differences: dorsal sepal ovate, acuminate, denticulate, reflexed at the apex, 2.6–2.7 mm long (vs. elliptic, subacute to obtuse, abruptly acuminate, margins smooth, 2.75 mm long); lateral sepals ovate, acuminate, denticulate, 2.5–2.6 mm long (vs. glabrous, ovate, acute, 3 mm long); petals transversely bilobed with narrowly triangular, falcate, ciliate lobes and a small triangular midlobe (vs. transversely bilobed with narrowly oblong, obtuse, subequal lobes, without a midlobe); lip blades narrowly ovate, acute, with a filiform, pubescent, inflexed appendix hidden in dorsal view (vs. ovate blades with rounded ends, appendix filiform, reflexed, and exposed in dorsal view).

*L. nasariana* also shows affinity with *L. trifurcata* Luer & R. Escobar, another member of L. subsect. Breves, but *L. nasariana* has succulent, oblong-lanceolate leaves, 15–20 mm long (vs. coriaceous, ovate leaves, ca. 35 mm long); shorter ramicauls of 18–23 mm with 4–5 lepanthiform sheaths (vs. longer ramicauls of 50–60 mm with 7 sheaths); and congested racemes up to 18 mm long, appressed to the abaxial surface of the leaf (vs. subcongested racemes only 3–5 mm long, borne behind the leaf). Floral differences are equally marked: in *L. nasariana*, the dorsal sepal is ovate, denticulate, 2.6–2.7 mm long (vs. subelliptic, glabrous, 6.5 mm long); the lateral sepals are 2.5–2.6 mm long, ovate, denticulate, connate for 1 mm (vs. narrowly ovate, 6.5 mm long, margins denticulate, only barely connate at the base); the petals are transversely bilobed, with narrowly triangular, falcate lobes and a marginal triangular midlobe (vs. deeply trilobed, with three similar, diverging, narrowly triangular lobes, including a long central lobe 2 mm in length); and the lip has narrowly ovate blades with a filiform, pubescent, inflexed appendix concealed in dorsal view (vs. lobes deeply bifid, falcate, enclosing the column, with a recurved, external appendix visible dorsally).

### ﻿Distribution model

**Figure 3.**
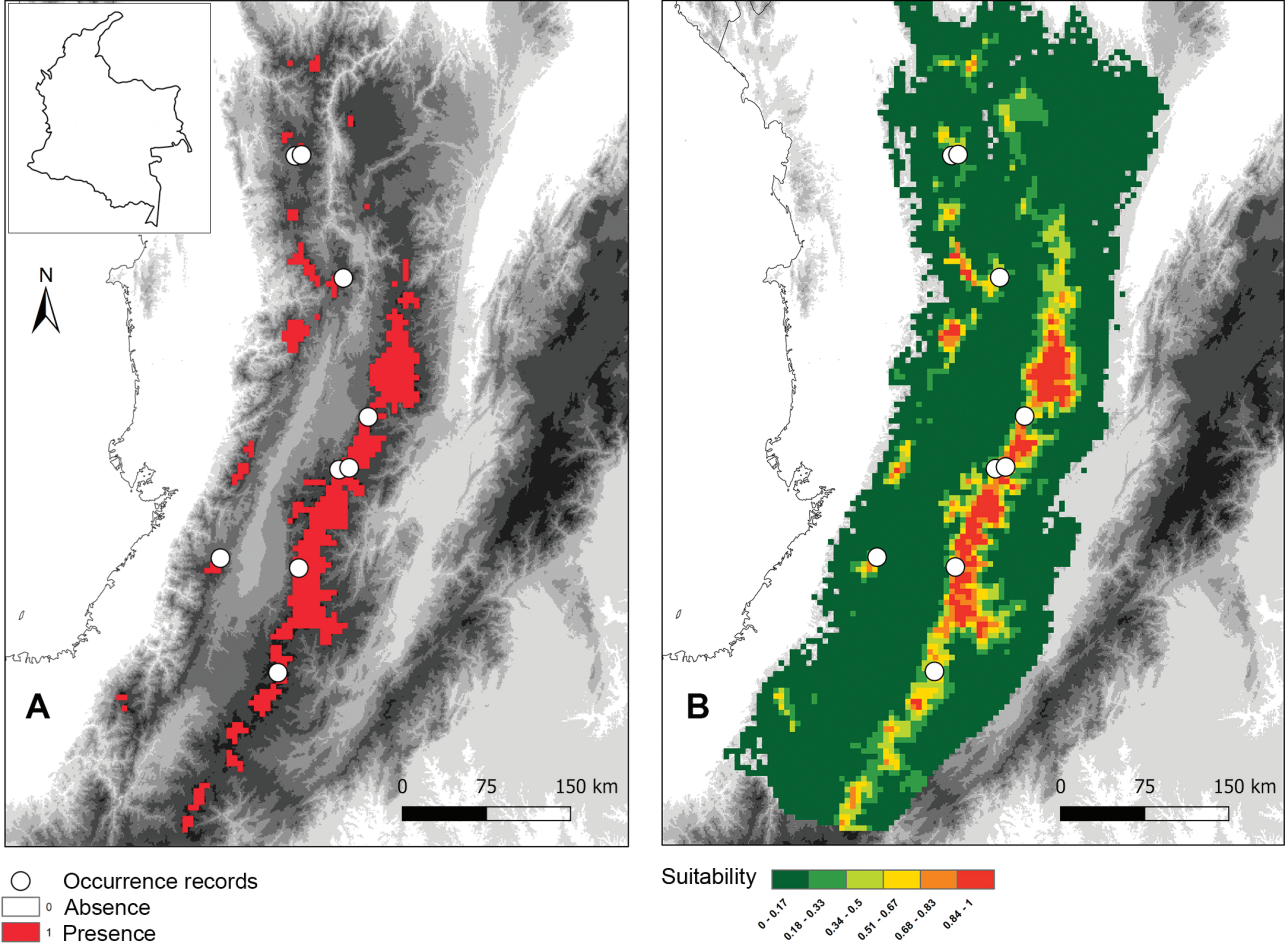
Current climate suitability model of *Lepanthes nasariana* J.S.Moreno & Hazzi, based on Maxent. A. Binary presence–absence map derived from the minimum training presence threshold, where red indicates areas predicted as suitable (presence = 1) and white indicates unsuitable areas (absence = 0); B. Continuous habitat suitability map showing logistic output values from 0 (unsuitable, dark green) to 1 (highly suitable, orange). White circles mark known occurrence records used to train the model.

The distribution model for *Lepanthes nasariana* exhibited high predictive performance, achieving an AUC_mean of 0.94 (SD = 0.042), well above random expectations. The model identified suitable habitat primarily in high montane and páramo ecosystems within the Central and Western Cordilleras of the Colombian Andes, at elevations of 2,500–3,200 m a.s.l. (Fig. 3A, B).

**Figure 4.**
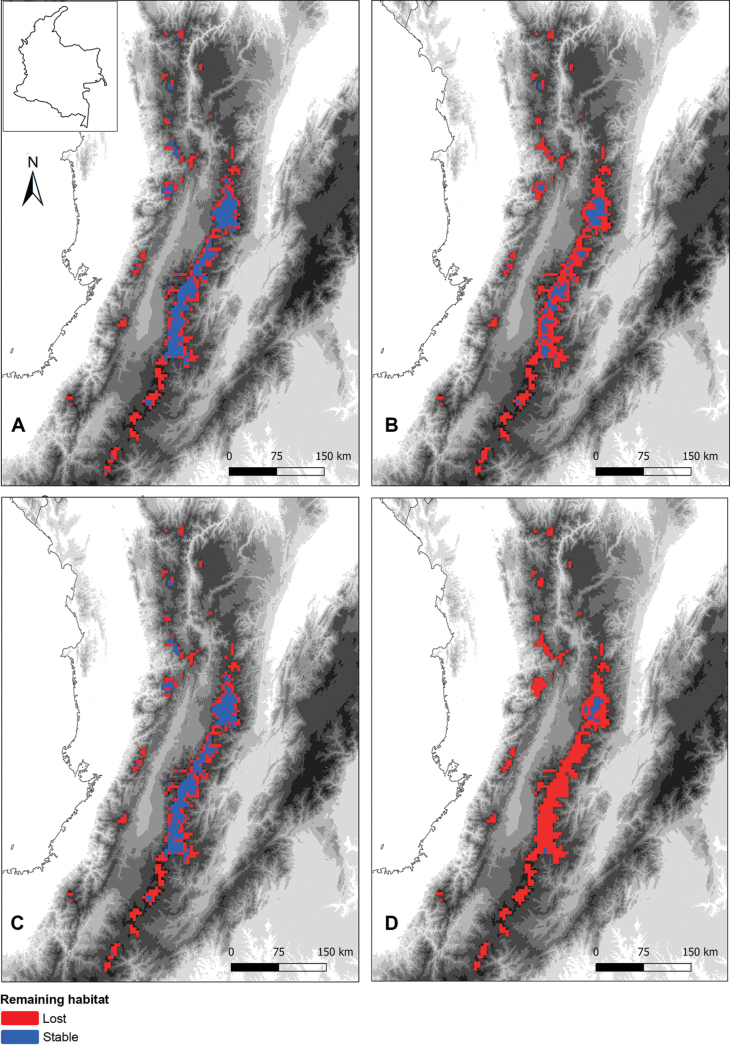
Projected changes in the potential distribution of *Lepanthes nasariana* J.S.Moreno & Hazzi, under future climate scenarios. **A.** SSP2-4.5 scenario for 2070; **B.** SSP5-8.5 scenario for 2070; **C.** SSP2-4.5 scenario for 2090; **D.** SSP5-8.5 scenario for 2090. Blue indicates stable habitat, red indicates habitat loss. Each map represents the ensemble average of multiple GCMs under intermediate (SSP2-4.5) and high (SSP5-8.5) greenhouse gas emission pathways.

Currently, *L. nasariana* occupies an estimated 12,775 km^2^ of suitable habitat. However, under future climate scenarios, significant habitat loss is projected. By 2061–2080, the SSP2–4.5 scenario predicts a 52% reduction in suitable habitat, leaving only 6,125 km^2^. The SSP5–8.5 scenario projects an even more severe reduction of 79%, reducing the suitable habitat to 2,625 km^2^. Further into the future, by 2081–2100, the SSP2–4.5 scenario estimates a slight recovery to 5,825 km^2^ (a 54% reduction from the current extent), while the SSP5–8.5 scenario predicts an almost complete collapse, with just 525 km^2^ remaining—equivalent to a staggering 96% loss of suitable habitat compared to present conditions (Figs 3, 4). The drastic reduction, particularly under the SSP5–8.5 high-emission scenario, suggests that by 2090, *L. nasariana* may only persist in two small refugia within the Nevados and Puracé National Parks of the Central Cordillera.

Uncertainty in the projections was quantified through coefficient of variation maps (Suppl. material 1: fig. S1), which showed higher variability in GCM predictions for the northern parts of the Western and Central Cordilleras. Conversely, projections for the central and southern regions displayed low variation, indicating greater confidence in the model predictions for these areas. Clamping maps from Maxent confirmed that no significant extrapolation occurred between present and future conditions, ensuring the robustness of the projections.

### ﻿Conservation status assessment of *Lepanthes nasariana* under present and future conditions

#### ﻿Present preliminary assessment

The current geographic distribution of *Lepanthes nasariana* reveals an extent of occurrence (EOO) of 27,502 km^2^ and an area of occupancy (AOO) of 12,775 km^2^. These values exceed the thresholds required to trigger Criterion B. Therefore, under current conditions, the species qualifies as Least Concern (LC).

Nonetheless, field observations indicate that *L. nasariana* is restricted to fewer than ten fragmented locations in the Western and Central Andes of Colombia. These populations are largely isolated and occur within forest remnants under anthropogenic pressure, including agriculture, cattle grazing, and forest degradation. While these conditions do not meet quantitative thresholds for Criterion B, they highlight the species’ ecological vulnerability and the need for monitoring and site-level protection.

#### ﻿Future projections

Climate-based SDMs projected to 2090 under SSP2–4.5 and SSP5–8.5 indicate a drastic contraction in suitable habitat, with a range-wide loss of up to 96%, leaving only two small refugia (Puracé and Nevados National Parks). This projected reduction suggests that *L. nasariana* would meet thresholds for Critically Endangered (CR) under Criterion A3(c), which considers projected declines of ≥ 80% due to habitat loss. Given the lack of species-specific generation length data, these projections should be interpreted as precautionary, exploratory assessments rather than definitive categorizations under Criterion A.

In addition, stochastic risk analyses based on SDM outputs indicated a high probability of extinction within 100 years. However, because these analyses do not constitute formal population viability analyses (PVAs), they cannot be considered a valid application of Criterion E under the IUCN framework. Instead, they should be viewed as complementary evidence underscoring the urgency of conservation interventions in light of climate-driven habitat loss.

#### ﻿Conclusion

Presently, *Lepanthes nasariana* does not qualify as threatened under Criterion B, given its current EOO and AOO. However, climate projections indicate severe future habitat contraction, consistent with thresholds for CR under Criterion A3(c) when interpreted within a precautionary framework. While our extinction risk modeling cannot formally support Criterion E, it highlights the likelihood of future population collapse if climate trajectories follow high-emission pathways. Together, these findings emphasize the importance of proactive conservation action and frequent reassessment of the species’ status.

## ﻿Discussion

This study documents the discovery of *Lepanthes nasariana*, a narrowly distributed orchid species endemic to the highlands of the Colombian Andes, whose extinction risk is already alarmingly high. Our models project that under the high-emission SSP5–8.5 scenario, the species will lose approximately 96% of its current suitable climatic habitat by 2090, with remnant populations restricted to small climatic refugia in Nevados and Puracé National Natural Parks. Under present conditions, the species qualifies as Least Concern (LC), given its current extent of occurrence (27,502 km^2^) and area of occupancy (12,775 km^2^), which are well above thresholds for Criterion B. However, such a drastic future range contraction suggests that the species would meet thresholds for Critically Endangered (CR) under Criterion A3(c) when interpreted within a precautionary and exploratory framework, as these projections are based on habitat loss rather than generation-length-specific reductions. We refer to this phenomenon—where a species’ future viability is already severely compromised at the moment of its scientific description—as the “Nasar Effect,” inspired by “Chronicle of a Death Foretold” (García Márquez 1983).

The case of *L. nasariana* illustrates a broader and increasingly common pattern in high-elevation tropical ecosystems. Global warming is projected to cause substantial range contractions in many high-Andean plants, particularly those restricted to páramos and upper montane forests. These ecosystems—among the most threatened in the Andes—are expected to undergo severe contraction or disappearance by the mid-21^st^ century under multiple climate scenarios in Colombia (Alarcón Hincapié and Pabón Caicedo 2013) and across the tropical Andes (Tovar et al. 2022). Such changes reflect not only species-level vulnerabilities but also ecosystem-wide shifts, as entire high-mountain communities migrate upward until they have nowhere left to go (Pounds et al. 1999; Manes et al. 2021; Mata-Guel et al. 2023). High-Andean flora, often characterized by small range sizes, ecological specialization, and dependence on specific microclimatic conditions (Hollenbeck and Sax 2024), is particularly susceptible to these climate-driven shifts.

Within orchids, the genus *Lepanthes* is emblematic of this vulnerability: approximately 70% of Colombian species are endemic and primarily restricted to montane regions (Moreno et al. 2020; Moreno 2024), and many exhibit narrow elevational ranges and strict ecological requirements (Crain and Tremblay 2014). Our findings are consistent with studies showing that similar neotropical montane epiphytes have limited capacity to survive outside their narrow climatic envelopes and low potential to track suitable conditions upslope under rapid warming (Hollenbeck and Sax 2024). These patterns are not unique to orchids—numerous Andean plant groups, from *Espeletia* Bonpl. (frailejones) to *Polylepis* Ruiz & Pav. woodlands, face similar risks under projected warming, making climate change one of the primary drivers of future biodiversity loss in the tropical Andes (Valencia et al. 2020; Caballero-Villalobos et al. 2021).

From a conservation perspective, our results underscore the urgency of adopting integrated strategies that combine in situ and ex situ measures. Protecting and restoring suitable habitats, establishing ecological corridors to facilitate altitudinal migration, and identifying and safeguarding potential climate refugia are critical for high-Andean orchids and other vulnerable flora. Ex-situ measures such as seed banking, cultivation in botanical gardens, and research into reproductive biology, genetic diversity, and population dynamics will be vital for building resilience and adaptive capacity (Liu et al. 2015; Corlett 2023). For species with extreme habitat loss projections such as *L. nasariana*, reciprocal transplant experiments could provide empirical data on climatic tolerances and inform prioritization of refugia (Morelli et al. 2016; Keppel et al. 2024).

We recognize, however, that species distribution models (SDMs) have inherent limitations. As correlative models, they assume that current climate–distribution relationships will hold under future conditions and that climate is the dominant driver of distribution at the scales analyzed. They may not fully account for microclimatic heterogeneity, dispersal limitations, or biotic interactions (e.g., pollinators, mycorrhizal fungi), nor for non-climatic threats such as habitat destruction. Although we used continuous suitability models and downscaled climatic data to better reflect fine-scale variation in the Andes, caution is warranted when interpreting projections, particularly for species with highly specialized niches and few occurrence records (Hollenbeck and Sax 2024). Future research should integrate field-based validation (e.g., transplant experiments), joint distribution models incorporating mutualistic partners, and land-use change projections to refine predictions and reduce uncertainty.

In conclusion, *Lepanthes nasariana* exemplifies how newly discovered species in biodiversity hotspots can already be on a trajectory toward extinction due to climate change. Much like Santiago Nasar’s fate in García Márquez’s novel, the species’ projected decline appears inevitable under current trends—but unlike Nasar’s story, there is still time to act. The challenge for conservation biology is to apply the precautionary principle, prioritize conservation planning and modeling under realistic to worst-case climate trajectories, and implement targeted conservation actions before the “death foretold” becomes reality for *L. nasariana* and countless other high-Andean plants.

## Supplementary Material

XML Treatment for Lepanthes nasariana

## ﻿Additional information

### Use of AI

AI-assisted writing support (ChatGPT, OpenAI) was used to improve the English grammar, structure, and clarity of the manuscript. All content was conceived, written, and verified by the authors, who take full responsibility for its accuracy and interpretation.

### Author ORCIDs

Juan Sebastián Moreno https://orcid.org/0000-0003-4325-1232

Angie Tatiana Herrera Cobo https://orcid.org/0009-0005-1100-9056

Rubén Darío Palacio https://orcid.org/0000-0001-9029-3094

Nicolas A. Hazzi https://orcid.org/0000-0002-7657-8963

### Data availability

All of the data that support the findings of this study are available in the main text or Supplementary Information.

## Supplementary materials

Supplementary material 1 Supplementary figure S1 Data type jpg Explanation note Uncertainty in ecological niche models for Lepanthes nasariana J.S.Moreno & Hazzi under current and future climate scenarios, based on the coefficient of variation (CV) across 15 Maxent replicates. Green indicates higher uncertainty, while blue indicates greater model agreement. A SSP2-4.5 scenario for 2070 B SSP5-8.5 scenario for 2070 C SSP2-4.5 scenario for 2090 D SSP5-8.5 scenario for 2090. https://binary.pensoft.net/file/1469556This dataset is made available under the Open Database License (http://opendatacommons.org/licenses/odbl/1.0/). The Open Database License (ODbL) is a license agreement intended to allow users to freely share, modify, and use this Dataset while maintaining this same freedom for others, provided that the original source and author(s) are credited.Juan Sebastián Moreno, Angie Tatiana Herrera Cobo, Rubén Darío Palacio, Nicolas A. Hazzi
