# Supplementary figures and images for: ﻿Chronicle of a death foretold: Lepanthes nasariana (Orchidaceae, Pleurothallidinae), a newly described high-Andean orchid facing a worst-case climate change scenario
Source: PhytoKeys. 2025 Nov 19;266:219–40. doi: 10.3897/phytokeys.266.161410 (PMC12658440; doi:10.3897/phytokeys.266.161410)

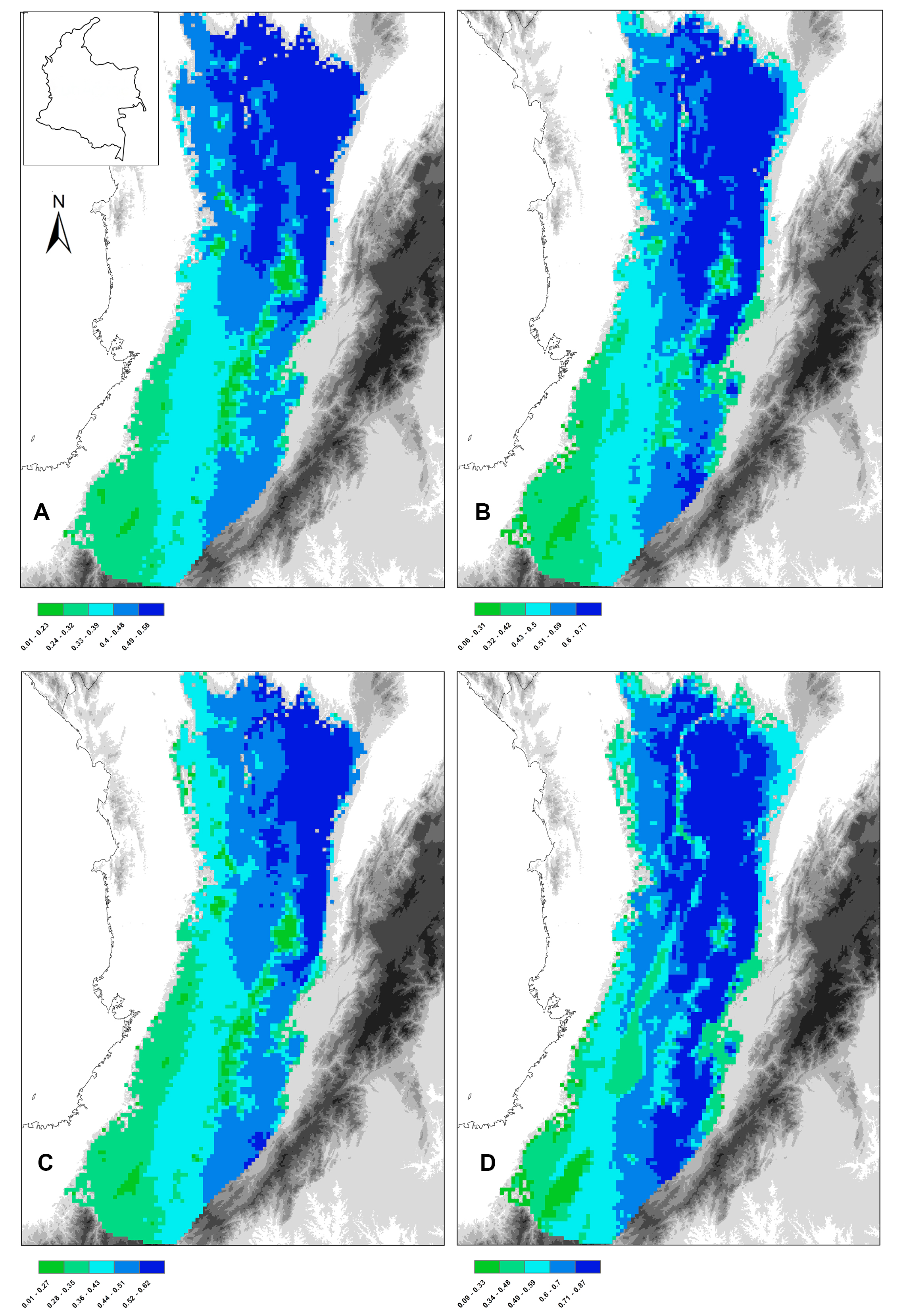

Supplement: Supplementary material 1 — Supplementary figure S1 [file phytokeys-266-219_article-161410__-s001.jpg]
